# Normalization of Elevated Blood Pressure After Anterior Cervical Discectomy and Fusion

**DOI:** 10.7759/cureus.25979

**Published:** 2022-06-15

**Authors:** Robert M Chory, Jason Craver, Ryan Cone, Susan Chory

**Affiliations:** 1 Obstetrics and Gynecology, Edward Via College of Osteopathic Medicine (VCOM) Auburn, Birmingham, USA; 2 Neurology, Edward Via College of Osteopathic Medicine (VCOM) Auburn, Birmingham, USA; 3 Education, Franklin Hospital, Floral Park, USA

**Keywords:** neuro-surgery, postoperative blood pressure, decompressive laminectomy, anterior cervical discectomy fusion, idiopathic hypertension

## Abstract

Perioperative change to the autonomic nervous system (ANS) in spine surgery is an uncommon occurrence but has a wide range of possible presentations including blood pressure, heart rate, and heart rate variability changes collectively referred to as dysreflexia. Increased sympathetic tone and decreased vagal output are believed to be the underlying causes of these autonomic manifestations and pose an important question as to effective treatment of these dysfunctions. Spinal nerve root decompression has shown to be a valuable tool in normalizing autonomic tone by increasing parasympathetic output, most notably to the cardiovascular system, leading to the resolution of the aforementioned cardiovascular complications. Here we report a patient with elevated blood pressure with complaints of upper extremity paresthesias. MRI showed nerve root compression, and anterior cervical discectomy was performed. Post-operatively the patient had a decrease in both systolic and diastolic blood pressure which was maintained two months after surgery and allowed for discontinuation of one anti-hypertensive medication.

## Introduction

The autonomic nervous system (ANS) consists of a collection of afferent and efferent fibers located throughout the body which allows for immediate response to stimuli. Any cause of insult to these fibers either through trauma, impingement, or other causes leads to an imbalance between sympathetic and parasympathetic tone leading to characteristic features [1]. Clinical presentation is largely affected by the level of impingement/injury, however, some common manifestations have been observed. Excess sympathetic tone from spinal cord insult or nerve root impingement most commonly presents with elevated blood pressure and heart rate, with less common findings including urinary retention and respiratory distress. Isolated parasympathetic tone due to spinal cord injury is less common than increased sympathetic tone alone but when present, typically manifests as a spinal shock, a life-threatening condition from loss of sympathetic output [1-3]. Unopposed parasympathetic tone due to spinal shock presents with hypotension, bradycardia, flaccid paralysis, and respiratory distress. Priapism was also a common finding in cases of unopposed parasympathetic output [1-3].

Dysautonomia is the abnormal alteration of the ANS due to chemical or structural neurological insult and is broadly classified into traumatic and atraumatic causes. Traumatic nerve compression has a wide variety of presentations based on the position, speed, and location at the time of injury. While the presenting cause of atraumatic spinal cord or nerve root compression widely varies as well, the underlying cause is more routine in presentation and is typically caused by herniated discs, arthritic changes of the spine, facet joint narrowing, or spinal stenosis [3,4]. Management of atraumatic spinal cord insult will depend on the degree and duration of stenosis as well as severity of symptoms but consists of more supportive measures initially, such as non-steroidal anti-inflammatory medication or epidural steroid injection, and surgical management later if conservative measures fail. Commonly used surgical techniques for spinal or nerve root decompression include laminectomy and discectomy both with and without fusion or artificial disc replacement [3-5]. Success rates of these procedures range from 66%-77% at 10-year follow-up for correction of lateral recess stenosis or spinal stenosis and herniated disc respectively [3-5]. Because of the well-described success, these procedures have had in reducing radiculopathy in patients with structural nerve insult, they have become a mainstay of treatment. However, a variety of additional benefits provided by spinal decompression surgery on blood pressure and heart rate have also been described and are current area of research.

## Case presentation

A 56-year-old white male presented for evaluation of numbness and tingling in his right hand. He stated he first noticed the pain while golfing four months earlier, but was able to manage the pain with acetaminophen and stretching. He has a past medical history of elevated blood pressure managed with metoprolol and lisinopril for two years. He states his blood pressure when measured at home is usually around 140/95. A blood pressure reading in the office showed a value of 142/91 on two separate readings. On physical exam decreased sensation was noted over the lateral forearm and proximal hand with no thenar muscle atrophy. The range of motion of the wrist, elbow, and shoulder was 5/5 bilaterally with minimal pain. A positive Hoffman sign was present on the right side, but Tinel and Phalen tests were negative bilaterally. Anterior and lateral cervical X-rays were taken showing moderate arthritic changes in the cervical spine with no obvious deformities, including lack of vertebral fracture and spondylolisthesis. An MRI of the cervical spine was ordered showing moderate lateral recess stenosis at the C5-C6 level. The patient was counseled on their options for management and chose to pursue an epidural nerve block before considering surgical intervention.

One month after the epidural block was performed, the patient returned with the complaint of reoccurring pain and consented to surgical decompression of the impinged nerve. A blood pressure reading taken pre-operatively before induction of anesthesia, in the hospital setting, showed a value of 141 mmHg systolic and 90 mmHg diastolic. The patient underwent anterior cervical discectomy and fusion (ACDF) at the C5-C6 level without complication. During operation, a 12mmHg and 9mmHg decrease in systolic and diastolic blood pressure respectively was noted at the time of nerve root decompression. The patient remained stable and no treatment or intervention was required at this time. The patient was discharged the same day with instructions not to perform a strenuous activity and acetaminophen for pain control per the patient’s request. Blood pressure and surgical scar were evaluated one week and four weeks after the operation revealing a well-healing scar and blood pressure values of 129/86 and 128/86 respectively. The patient reported he had stopped taking the metoprolol previously prescribed for his elevated blood pressure at the suggestion of his primary care physician due to an episode of hypotension, but has continued to take lisinopril. One additional follow-up with the patient at two months post-operation showed minimal scarring at the incision site, resolution of “90 percent” of his pain, and a blood pressure value of 128/87.

## Discussion

Primary hypertension is one of the most commonly diagnosed medical conditions and is typically managed with pharmacologic therapy. The vast majority of cases of hypertension are related to diet and poor lifestyle choices, however rarer causes of elevated blood pressure, such as spinal nerve root impingement, should be included in the differential [4]. There are many theories for the cause of elevation in both systolic and diastolic blood pressure in nerve root impingement, with the most widely accepted theory being the loss of parasympathetic tone leading to increased sympathetic tone [6]. Other theories state that chronic irritation of sympathetically innervated structures, such as the vertebral discs and dura mater, leads to increased firing of sympathetic nerves and ultimately elevated blood pressure. A higher incidence of hypertension has been noted in patients with compression at the C3-C4 level when compared to other single-level compressions, believed to be attributed to stimulation of the superior cervical ganglion which is formed from nerve fibers from the C1-C4 levels [6]. This has provided tangible support for these theories, although more investigation as to the definitive underlying cause of nerve root compression causing hypertension is needed.

Patients with elevated blood pressure have historically experienced a decrease in both systolic and diastolic blood pressure following surgical decompression of nerve root impingement. This association has led to more investigation of the effects of nerve root compression on blood pressure. A review of the literature showed five major studies evaluating the relationship between nerve root impingement and hypertension, including a total of 964 patients with an average age of 55.42 years old [6]. Blood pressure values were recorded pre-operatively and either at post-op month 1, 3, 6 or 12. Results showed an average decrease of 8.34 mmHg, 13.06 mmHg and 6.18 for mean arterial pressure (MAP), systolic blood pressure and diastolic blood pressure respectively between pre-operative to post-operative recordings [6]. A similar result was seen in our patient with a described decrease of 12mmHg and 8 mmHg in systolic and diastolic blood pressure respectively from original visit to 2 month post-operative visit. Few prior studies report the discontinuation of anti-hypertensive medications after surgical correction, as seen in our case, and is an area for future research consideration.

Because of these results nerve root impingement should be considered as a possible cause of elevated blood pressure with management by cervical decompression. It should also be noted that while nerve root decompression surgery may significantly lower blood pressure it should not replace the current pharmacologic anti-hypertensive treatments and should only be performed in those who are surgical candidates regardless of blood pressure level. While a statistically and clinically significant decrease in blood pressure was reported after surgical decompression was performed more information is needed to explore this relationship, as well as the long term effects this drop in blood pressure has on typical hypertension related complications such as stroke, and decreased life span [4-6].

## Conclusions

Elevated blood pressure and other cardiovascular disease are one of the leading causes of morbidity in the United States and is most commonly caused by lifestyle choices such as poor diet and a sedentary lifestyle. Hypertension is typically managed solely with pharmacotherapy such as angiotensin-converting-enzyme (ACE) inhibitors and beta-blockers, however, surgical correction of neurologic structural disorders such as cervical nerve root impingement may also play a role. While surgical correction is currently not recommended as a treatment for patients with hypertension alone, there is evidence that those who have concurrent elevated blood pressure and nerve root impingement may achieve long-term relief of both neurologic and cardiovascular morbidities, with a small subset of patients being able to decrease or stop taking their anti-hypertensive medications altogether, as described in this case.
